# Assays for estimating HIV incidence: updated global market assessment and estimated economic value

**DOI:** 10.1002/jia2.25018

**Published:** 2017-11-22

**Authors:** Charles S Morrison, Rick Homan, Natasha Mack, Pairin Seepolmuang, Megan Averill, Jamilah Taylor, Jennifer Osborn, Peter Dailey, Neil Parkin, Stefano Ongarello, Timothy D Mastro

**Affiliations:** ^1^ Global Health, Population and Nutrition FHI 360 Durham NC USA; ^2^ FHI 360 Hanoi Vietnam; ^3^ HIV/HCV Department The Foundation for Innovative New Diagnostics (FIND) Geneva Switzerland

**Keywords:** HIV incidence, laboratory assays, surveillance, HIV, surveys, HIV testing

## Abstract

**Introduction:**

Accurate incidence estimates are needed to characterize the HIV epidemic and guide prevention efforts. HIV Incidence assays are cost‐effective laboratory assays that provide incidence estimates from cross‐sectional surveys. We conducted a global market assessment of HIV incidence assays under three market scenarios and estimated the economic value of improved incidence assays.

**Methods:**

We interviewed 27 stakeholders, and reviewed journal articles, working group proceedings, and manufacturers’ sales figures. We determined HIV incidence assay use in 2014, and estimated use in 2015 to 2017 and in 5 to 10‐years under three market scenarios, as well as the cost of conducting national and key population surveys using an HIV incidence assay with improved performance.

**Results:**

Global 2014 HIV incidence assay use was 308,900 tests, highest in Asia and mostly for case‐ and population‐based surveillance. Estimated 2015 to 2017 use was 94,475 annually, with declines due to China and the United States discontinuing incidence assay use for domestic surveillance. Annual projected 5 to 10 year use under scenario 1 – no change in technology – was 94,475. For scenario 2 – a moderately improved incidence assay – projected annual use was 286,031. Projected annual use for scenario 3 – game‐changing technologies with an HIV incidence assay part of (a) standard confirmatory testing, and (b) standard rapid testing, were 500,000 and 180 million, respectively. As HIV incidence assay precision increases, decreased sample sizes required for incidence estimation resulted in $5 to 23 million annual reductions in survey costs and easily offset the approximately $3 million required to develop a new assay.

**Conclusions:**

Improved HIV incidence assays could substantially reduce HIV incidence estimation costs. Continued development of HIV incidence assays with improved performance is required to realize these cost benefits.

## 1 Introduction

Measures of HIV incidence are important public health indicators that describe the intensity of the HIV epidemic, are used to evaluate the impact of prevention interventions, help policymakers better allocate resources, and assist in identifying sites for HIV prevention trials. Despite the importance of having robust HIV incidence data in the fight against the HIV epidemic, determining the best strategy for measuring it remains a challenge due to the limitations of available methods. Currently, incidence estimates are obtained through either statistical modelling from survey prevalence data, direct measurement in longitudinal cohort studies, or laboratory‐based biological assays from cross‐sectional surveys. Incidence estimates from models based on prevalence survey data provide incidence estimates for the past and are subject to bias and wide ranges of uncertainty 1. Longitudinal cohorts are costly, subject to participation bias, and represent the incidence in the study population. A reliable laboratory assay capable of measuring recent HIV infection could save time, human resources and cost compared to other methods for obtaining HIV incidence estimates by providing time‐sensitive incidence (i.e. at the time of the survey) and therefore more actionable information for efficient and effective programme management.

One example of use of HIV incidence assays (HIAs) for this purpose are the ongoing population‐based HIV impact assessments (PHIAs) conducted by the President's Emergency Plan for AIDS Relief (PEPFAR), to obtain national incidence estimates in 14 countries, primarily in sub‐Saharan Africa 2. HIAs also hold the potential to improve individual patient management, including counselling, treatment strategies, and contact tracing.

Several HIAs have been developed based on the principle that the antibody response to HIV matures over time to distinguish recently acquired from long‐standing infection based on quantitation of anti‐HIV antibody response. These include assays such as the BED CEIA (capture enzyme immunoassay) and the limiting antigen (LAg) avidity assay, as well as commercial diagnostic kits modified to measure HIV incidence (e.g. Bio‐Rad HIV‐1/2 EIA Avidity assay) 3.

HIA test performance is characterized by two key parameters: mean duration of recent infection (MDRI) and the false‐recent ratio (FRR). MDRI is the average time spent “recently” infected within some time “*T*” after infection. FRR is the probability that an individual who is infected for longer than *T* will produce an erroneous “recent” result 4. Sample sizes and associated implementation costs required for precise incidence estimation are driven by the prevalence and incidence in the testing population, and these performance characteristics (MDRI, FRR). HIAs with improved test performance – namely longer MDRI and lower FRR – could substantially reduce survey implementation costs. Other factors including HIV subtype and ART coverage also impact HIA performance and consequently, sample size requirements 5.

The Foundation for Innovative New Diagnostics (FIND) has been supporting, coordinating, and managing the activities of multiple groups working on HIAs, including development of Target Product Profiles, an updated HIA market landscape, and coordination of new assay development and evaluation. As part of this effort, a description of HIA use cases has been developed (Table S1) 6.

In 2009, FHI 360 conducted a global landscape and market assessment of HIAs 7, 8. In 2015, advances in HIA technology and recent infection testing algorithms (RITAs), suggested that an updated market assessment of HIAs could help focus future efforts. The aim of this market assessment was to measure actual 2014 HIA use, estimate 2015 to 2017 annual HIA use, to project future demand (5 to 10 years) for HIAs under various market scenarios, as well as to estimate the economic cost and benefits of developing HIAs with improved performance.

## 2 Methods

### 2.1 Market assessment

We interviewed seven key stakeholders and 22 other experts (Table S2) about their insights into market demand for current and/or improved HIAs over the next 5 to 10 years. Interviews were conducted by telephone using an interview guide with questions adapted to the category of respondent (i.e. user, manufacturer, funder). Additional sources included journal articles and reports on HIV surveillance, laboratory and field‐based experiences with HIV incidence estimation, and proceedings of international working groups and normative bodies.

We leveraged the expertise of stakeholders and experts to calculate 2014 HIA use based on HIAs used (i.e. number of samples tested) or sold. We used data provided by the manufacturers for some of the HIAs (BED, LAg). For other HIAs (for which we had no manufacturers’ sales data) we used data from the interviews. We estimated 2015 to 2017 global use by country and use case. To address market demand in 5 to 10 years, we considered the following three demand scenarios:

- Scenario 1 – no change in HIA/RITA technology – calculated as the average 2015 to 2017 annual use ±25% for low and high bounds around the “no change” estimate (based on expert feedback of expecting no change in HIA/RITA use with no change in technology/performance).
- Scenario 2 – a moderate improvement in HIA/RITA performance (e.g. in antiretroviral‐treated patients, persons infected with HIV‐1 subtype D viruses, longer MDRI, etc.) – would result in a modest/moderate increase in HIA use. This was calculated as the mean of 2015 to 2017 planned annual use (with China's 2014 case‐based surveillance (CBS) figures added back) and increased by 35% (estimate of modest/moderate increased use) ±25% for low and high bounds.
- Scenario 3 – a “game‐changing” HIA/RITA becomes available – was split into two scenarios:
  - Scenario 3a: Changes in technology result in HIAs/RITAs being used in routine confirmatory testing to inform individual patient management. This estimate was based on the number of incident HIV cases projected by UNAIDS in 2020 (500,000) under the strategy for fast tracking the AIDS response for low and middle‐income countries 9. A lower bound is 65% of annual HIV‐positive diagnoses assuming not all countries introduce HIAs/RITAs in confirmatory testing; an upper bound is 135% of annual diagnoses, assuming that not all planned prevention interventions are implemented over 5 to 10 years.
  - Scenario 3b: Technology changes result in a combined diagnostic/recency rapid test that becomes standard worldwide. The limit for scenario 3b is the number of HIV tests conducted each year for diagnostic/screening purposes, based on UNAIDS reports for low‐middle income countries (figures for high‐income countries unavailable). A lower bound is 65% of total diagnostic tests used globally assuming HIA use in screening and diagnostic testing are not introduced in all countries within 5 to 10 years.

### 2.2 Cost to develop an assay based on a new HIA biomarker

We estimated cost to develop a highly complex multiplex assay based on a new biomarker(s) concept ready for technology transfer to an ISO 13485‐certified diagnostic company for full development at $3.2 million (Table S3). The estimate considered four phases of development (technical feasibility, development, manufacturing transfer, and external evaluation) and the parties involved (manufacturer, academic partner, and external evaluator). For each phase, full‐time equivalent labour required (valued at $225,000 annually) for 11.4 person‐years of work over a 36‐month period was estimated along with additional costs for development and quality assurance specimens, equipment, and specimens and kits for external validation. These cost estimates (details in Table S7) are based on the diagnostic industry experience of one of the authors (PD) and were verified with two additional diagnostic industry experts. If substantial research and development work were still needed at technology transfer, the development phase would be 1.5 to 2.0 times longer with a corresponding increase in development phase costs.

### 2.3 Economic value of an improved HIA

We estimated the economic value of an improved HIA in terms of public health dollars saved as a result of more efficient surveillance efforts, and improved evaluation, decision making, and spending on HIV prevention efforts. For population‐based surveys, the anticipated sample size was calculated based on the number of recent HIAs obtained from the market assessment and expected HIV prevalence in the population. For sentinel (including key populations such as sex workers and men having sex with men) surveys, we retrieved raw data from the HIV/AIDS Surveillance Data Base 10, examining incidence studies from 2009 to 2014. We cleaned and removed duplicate data to estimate the annual expected number of surveys and corresponding sample size.

We assessed potential reductions in survey sample sizes if the HIA had an extended MDRI 11. We based this on South African national surveillance data for women aged 15 to 24 years, an FRR of 0.25%, but extended MDRI from 130 days (current MDRI for a RITA of LAg avidity OD‐n <1.5 and viral load (VL) >1000 copies/mL for subtype C) to 154 (using nucleic acid screening to capture acute infections, detectable 24 days before seroconversion), 240 and 280 days.

Our baseline analysis assumed that only 80% of potential sample size reductions could be realized, as other objectives besides incidence estimation could drive sample size (e.g. desire for stratified estimates, estimates of other parameters such as VL suppression). We then also considered 50% and 100% of the potential reduction as worst‐ and best‐case scenarios.

National and key population surveillance costs were calculated based on budgets from recent bio‐behavioural surveys conducted in Ghana and Botswana by FHI 360. Detailed survey budgets (M. Merrigan, personal communication) (Table 1) were examined to identify the variable portion of total survey costs (i.e. sensitive to sample size) and those linked to population based versus key population surveillance surveys. Fixed costs were estimated at $210,000 for population‐based surveys and $110,000 for key population surveillance surveys; variable costs were estimated on a per sample basis ($180 and $70 for population‐based and key population surveillance surveys, respectively) (Table S4). As sample size requirements were reduced (based on longer MDRI), the variable cost of anticipated surveys was reduced and annual net savings across planned surveys computed.

**Table 1:** Surveillance survey sample sizes and costs by use case and time frame

	Use case description	Use next 2 to 3 years	Use 5 to 10 years Scenario 2	
	Total annual sample size	Total annual cost (USD)^a^	Total annual sample size	Total annual cost (USD)^a^
Population‐based surveys	168,368	31,881,186	227,296	42,488,351
Key population Surveillance surveys	105,000	9,220,000	141,750	12,447,000
Total	273,368	41,101,186	369,046	54,935,351

a Total annual costs based upon: (number of surveys x fixed costs per survey) + (survey sample size × variable cost per subject); see Table S4 for cost details by type of survey.

### 2.4 Estimated public health impact of an improved HIA

To estimate the maximum potential public health impact of reprogramming annual savings from an improved HIA, HIV prevention interventions were ranked with respect to the estimated annual cost per HIV infection averted 12, 13 and funds allocated to interventions with the lowest annual cost (mass media campaigns, peer education and sexually transmitted infections (STI) treatment for sex workers, at $58 per infection averted, and male circumcision at $300/infection averted) until “full coverage” was achieved. This was dependent on implementation of these interventions being consistent with the programmes from which effectiveness data are estimated, and the interventions being successful in reaching their target populations.

As a conservative estimate, the average annual discounted lifetime cost of HIV/AIDS treatment was used to convert infections averted into a monetary figure (not including savings to caregivers, increased productivity of persons who avoid HIV infection, or funeral costs averted). Using the direct treatment costs (e.g. drugs, lab monitoring, and management of opportunistic infections), we narrowly focused the public health impact on the health care system. Recent estimates of discounted annual lifetime costs of HIV/AIDS care and treatment for sub‐Saharan Africa are $200 to 1200/year with a median value of $612 11, 14, 15, 16, 17. We used this median value to estimate the annual public health impact associated with an improved HIA as HIV infections are averted.

### 2.5 Comparison of surveillance cost savings to development costs

We relaxed the assumption that 80% of the potential sample size reduction would be obtained (base case) and compared the potential 2 to 3‐year annual cost savings as the percentage of sample size reduction decreases to the estimated costs of developing an improved HIA. The objective was to identify the threshold where costs of development exceed potential annual cost savings in the short‐term (e.g. if the payback period for developing the HIA required multiple years).

To extrapolate the potential public health benefit from the cost‐savings of HIAs with improved performance, we estimated how annual savings could be re‐programmed into improved prevention efforts. Data from a systematic review of HIV prevention cost‐effectiveness and the WHO‐CHOICE model results were used to translate potential annual cost‐savings into estimates of annual HIV infections averted, as savings are directed to the most cost‐effective HIV prevention activities 12, 13. Using the annual number of HIV infections averted and the discounted annual costs of HIV treatment 12, we estimated annual downstream cost‐savings from reduced treatment costs.

## 3 Results

### 3.1 Market assessment

We calculated total global HIA use for 2014 at 308,893 samples tested (Table 2). HIA use was highest in Asia, especially China, with 138,364 HIAs (45% of global use), followed by the Americas, Africa, and Europe. Most HIAs were used in CBS, followed by population‐based surveys for national surveillance, research evaluations of HIAs, key population surveillance, and assessing population‐level intervention impact. In 2014, the most used HIA remained the BED CEIA 18 at 47% (80% in China), followed by the LAg 19, and BioRad Avidity assays 20. Other HIAs included the Architect Avidity 3, 21, BioRad Geenius 22, 23, Glasgow Avidity 24, and the IDE‐V3 25, 26.

**Table 2:** HIA use (number of samples tested) in 2014 and estimated annual (2015 to 2017) use (samples to be tested) by use case and region

Region	National surveillance	Sentinel surveillance	Intervention impact	Case‐based surveillance	Research^a^	HIA evaluation	Unknown use	Total
2014 HIA use (number of samples tested) by use case and region								
Africa	25,300	750	0	0	0	0	7900	33,950
Americas	0	1350	6000	41,200	1200	4800	1500	56,050
Asia Pacific	0	8900	0	124,764	0	0	4700	138,364
Europe	0	929	0	22,600	0	0	1500	25,029
Other^b^	0	0	0	0	0	12,500	0	12,500
Unknown^c^	0	0	0	0	0	0	43,000	43,000
Total	25,300	11,929	6000	188,564	1200	17,300	58,600	308,893
Average estimated annual (2015 to 2017) HIA use (samples to be tested) by use case and region								
Africa	8889	2250	0	0	0	0	**‐**	11,139
Americas	0	1350	7500	29,733	1500	6000	**‐**	46,083
Asia Pacific	0	3500	0	7936	0	0	**‐**	11,436
Europe	0	1100	0	23,467	0	0	**‐**	24,567
Other^b^	0	0	0	0	0	1250	**‐**	1250
Total	8889	8200	7500	61,136	1500	7250	**‐**	94,475

a Identification of individuals with recent infection for research purposes.

b Other region – Consortium for the Evaluation and Performance of HIV Incidence Assays (CEPHIA) evaluations are conducted across all regions.

c Unknown region – Location sold not reported by manufacturer.

We estimated 2015 to 2017 global use at 94,475 annually (Table 2). Planned use was highest for the Americas (49%), followed by Europe, Africa, and Asia, with most of the decline resulting from China and the United States discontinuing HIA use for CBS in 2017. Most 2015 to 2017 planned use is for CBS (65%), followed by population‐based surveys, key population surveillance, assessing population‐level intervention impact, and research evaluations. Over 2015 to 2017, planned HIA use shifts from BED (15%) to LAg (40%) and BioRad (35%) avidity assays.

We estimated annual HIA use in 5 to 10 years under Scenario 1 (no change in technology) at 94,475 (Table 3). The largest number is for the Americas, followed by Europe and Africa, the majority for CBS. For Scenario 2 (moderately improved performance), we projected a modest increase in annual usage (286,031), highest in Asia (assuming China resumes HIA use for CBS), followed by the Americas, Europe, and Africa.

**Table 3:** Estimated annual HIA use (samples to be tested) 5 to 10 years in future by scenarios

	Estimated use	Lower bound	Upper bound
Scenario 1: No change in technology	94,475	70,856	118,094
Scenario 2: Moderately improved technology	286,031	214,523	357,539
Scenario 3a: Part of all HIV confirmatory testing	500,000^a^	325,000	675,000
Scenario 3b: Part of all HIV #6;screening/diagnostic testing b	180,000,000^a^	117,000,000	180,000,000

a Estimated use assumes that all confirmatory testing includes HIA use (scenario 3a) and that all diagnostic/screening testing includes HIA use (scenario 3b).

b Estimated for low and middle income countries only.

For Scenario 3a (game‐changing technology combining HIA/RITA with HIV confirmatory testing), based on the number of projected HIV incident cases in 2020, our estimate is 500,000 tests. Most HIAs would be used in Africa with the highest number of new cases. For Scenario 3b (more dramatic game‐changing technology with HIA/RITAs becoming part of all rapid screening/diagnostic tests), based on WHO estimates of HIV diagnostic tests used annually in low‐ and middle‐income countries 27 and extrapolating to 2020 to 2022, the estimate is approximately 180 million (Table 3). Use of additional tens of millions of tests is likely in high‐income countries.

### 3.2 Economic value of an improved HIA

Fourteen 2 PHIA‐type population‐based surveys are planned over the next 2 to 3 and 5 to 10 year periods with total annual sample size estimated at 168,368 and 227,297, respectively (Table 1). Individual sample sizes range from 5907 in Kaduna State, Nigeria (2 to 3 years) to 40,000 for a PHIA in Côte d'Ivoire, Tanzania and Cameroon (Table S5). The resulting costs of conducting these surveys ranges from approximately $1.3 to $7.4 million ($210,000 for fixed costs per survey plus variable costs of $180/subject) (Table S4). We estimate 17 key population surveys per year for the next 2 to 3 years with a total annual sample size of 105,000 persons. Based on stakeholder interviews, we've projected a 35% increase (see Methods) in the number of people surveyed in the next 5 to 10 years with annual survey costs from approximately $131,000 to $1.2 million, again depending upon expected sample size (fixed costs at $110,000/survey and variable costs of $71/subject).

Estimates of the impact of increasing the MDRI from 130 to 154, 240, or 280 days were based on previous work 11 computing the potential reduction sample size reductions achievable for longer MDRIs for both national and key population incidence estimation use scenarios. When decreasing the potential reduction to 80% of the maximum (see Methods), reductions in survey sample size were: 18.1%, 50.3%, and 57.7% for national surveillance and 16.6%, 47.5%, and 54.7% for key population/sentinel surveillance for the different MDRI values, respectively. We then estimated total annual cost savings by reducing the corresponding variable cost component of the surveys (top half of Table 4). Our base case result is that an improved HIA could reduce the *annual* cost of national surveillance and key population surveys from between $5 to 17 million in the next 2 to 3 years to $7 to 23 million in 5 to 10 years (corresponding to increased MDRIs of 154 to 280 days).

**Table 4:** Potential annual cost savings from reduced sample size requirements as MDRI increases from 130 days and potential annual HIV/AIDS treatment cost savings if surveillance survey savings invested in cost‐effective HIV prevention interventions, by use case and time frame (assuming 80% of potential sample size reductions are realized)

	Next 2 to 3 years			Next 5 to 10 years (Scenario 2)		
Use case description	154 days	240 days	280 days	154 days	240 days	280 days
Potential annual cost savings (USD) from reduced sample size requirements as MDRI increases from 130 days to indicated MDRI						
Population‐based surveys	4,388,336	12,195,209	13,989,335	5,924,253	16,463,532	18,885,603
Key population surveillance surveys	976,080	2,793,000	3,216,360	1,317,708	3,770,550	4,342,086
Total (range)	5,364,416 (3.4 to 6.7 million)	14,988,209 (9.4 to 18.7 million)	17,205,695 (10.8 to 21.5 million)	7,241,961 (4.5 to 9.0 million	20,234,082 (12.6 to 25.3 million)	23,227,689 (14.5 to 29.0 million)
Potential annual HIV/AIDS treatment cost savings (USD) if surveillance survey savings invested in cost‐effective HIV prevention interventions, with indicated MDRI						
Population‐based surveys	46,014,353	127,874,141	146,686,639	62,119,376	172,630,091	198,026,963
Key population surveillance surveys	10,234,789	29,286,294	33,725,479	13,816,965	39,536,496	45,529,397
Total	56,249,142	157,160,435	180,412,118	75,936,341	212,166,587	243,556,360

MDRI, mean duration of recent infection.

### 3.3 Estimated public health impact of an improved HIA

With the assumption that the savings from smaller required sample sizes for Population‐based and Key Population Surveillance surveys can be effectively redirected to the cost‐effective HIV prevention interventions, we estimate that between 91,911 to 294,791 HIV infections could be averted per year in the short term rising to 124,079 to 497,967 per year in the 5 to 10‐year period (Table S6) depending upon the increase in MDRI. Assigning a value of $612 (median annual discounted lifetime costs of HIV/AIDS treatment) to these infections yields a potential public health cost savings of between $56.2 to $180.4 million per year in the short term rising to $75.9 to $243.5 million per year in the 5 to 10‐year period (bottom half of Table 4) depending on the increase in MDRI.

### 3.4 Comparison of surveillance cost savings to development costs

**Figure 1:**
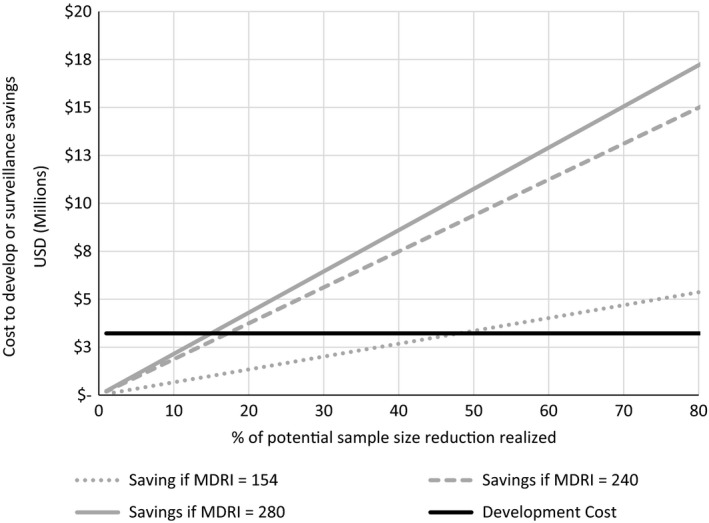
Cost savings of lengthened MDRI compared to development costs of improved HIA by % of potential sample size reduction realized. As long as sample sizes are able to be reduced by at least 48% of their potential for an MDRI of 154 days and for 17% to 15% of their potential for MDRIs of 240 and 280 days, the annual savings in the 2 to 3‐year period will more than offset the costs of new assay development. MDRI, mean duration of recent infection.

Our analysis shows that improved HIAs with longer MDRI are cost saving and only under extremely unfavorable circumstances would annual savings over the 2 to 3‐year period be unable to offset assay development costs (Figure 1). For example, only if less than 48% of potential cost saving are realized for an MDRI of 154 days (or less than 17.2% and 15% of MDRIs of 240 and 280 days, respectively) would development costs outweigh the reduction in survey costs. This would only occur if other surveillance survey objectives require keeping additional sample size (e.g. for sub‐group incidence estimation).

### 3.5 Estimated public health impact of an improved HIA

To estimate the maximum potential public health impact of reprogramming annual savings from an improved HIA (Table S6), HIV prevention interventions were ranked with respect to the estimated annual cost per HIV infection averted 12, 13 and funds allocated to interventions with the lowest annual cost (mass media campaigns, and peer education and STI treatment for sex workers, at $58/infection averted, and male circumcision at $300/infection averted) until “full coverage” was achieved. This was dependent on implementation of these interventions being consistent with the programs from which effectiveness data are estimated, and the interventions being successful in reaching their target populations.

As a conservative estimate, the average annual discounted lifetime cost of HIV/AIDS treatment was used to convert infections averted into a monetary figure (not including savings to caregivers, increased productivity of persons who avoid HIV infection, or funeral costs averted). Using the direct treatment costs (e.g. drugs, lab monitoring, and management of opportunistic infections), we narrowly focused the public health impact on the healthcare system. Recent estimates of discounted annual lifetime costs of HIV/AIDS care and treatment for sub‐Saharan Africa are $200 to 1200/year with a median value of $612 11, 14, 15, 16, 17. We used the median value to estimate the annual public health impact associated with an improved HIA as HIV infections are averted (Table S6).

## 4 Discussion

A cross‐sectional HIV incidence assay remains a high priority to provide cost‐ and time‐effective HIV incidence data to guide prevention programs. Considerable efforts have been made to evaluate the performance of various HIAs/RITAs, define use cases and associated target product profiles, and estimate future market demand 3, 7, 23. Normative guidance groups continue to convene stakeholders to discuss unresolved issues and provide guidance 6. Nevertheless, the market for HIAs remains relatively modest.

Market demand for HIAs over the next 5 to 10 years based on current technologies appears largely driven by CBS. Currently, many European and North American countries use HIAs for this purpose, as did China in 2014. Although China does not plan to use HIAs for 2015 to 2017, it is planning to reinstitute use in the future using a Chinese manufacturer, according to one stakeholder we interviewed. Several African countries (e.g. Senegal, Kenya) are interested in developing CBS systems and may want to employ HIAs. Additionally, improvements in HIA technology and performance could lead to additional use for national and key population surveillance, and for evaluation of public health interventions.

The market for HIAs would be substantially increased by the development of “game‐changing” HIAs/RITAs used to determine HIV recency in individuals. The Consortium for the Evaluation and Performance of HIV Incidence Assays (CEPHIA) has recently evaluated the BioRad Geenius (used as an HIV confirmatory test in the US) to determine if infection recency can be accurately determined by evaluating the different glycoprotein bands 23. If a recency test were available as part of HIV confirmatory testing and regulatory approval could be obtained (depending on test location and purpose) for determining HIV recency in individuals, HIAs could be used for individual patient management. Under such a scenario (3a), HIA use could increase significantly to approximately 500,000 annually.

An even larger demand increase could occur with availability of a rapid test that determines both HIV positivity and recency (a “diagnostic/recency” or “prevalence/incidence” test), such as that under development at CDC in conjunction with two manufacturers 28, 29. If this type of “game‐changing” assay has adequate performance characteristics for various HIV‐1 subtypes, is affordable, and if a recency indicator becomes standard for all rapid tests, a dramatic increase in HIA demand could result – upwards of 180 million tests annually over the next 5 to 10 years.

However, such a scenario faces several obstacles. First, it would have to pass technical/performance standards as an HIV diagnostic and may have to be part of a RITA. Adding VL testing to a rapid test could dramatically limit its use unless VL testing was available at point‐of‐care and highly affordable. Additionally, it would have to pass regulatory approval for the new indication of determining recency. Thus, significant barriers remain to realization of scenario 3b in the next 5 to 10 years.

We estimated the public health value of the potential gain from redirecting public health dollars saved from improvements in HIAs (scenario 2) used for surveillance towards increased investments in effective HIV/AIDS prevention efforts. The estimated public health impact (value of infections averted through prevention) is approximately 10.5 times greater than the estimated potential savings from decreased surveillance costs as the MDRI increases from 130 days. This may be an optimistic estimate as it assumes the most cost‐effective prevention interventions receive these funds and that these interventions are fully implemented. However, the conclusion of a net value gain would persist even if the cost to prevent an infection were to rise to $600 from our base estimate of $58. Also, because other indicators measured as part of national or key population surveys (e.g. viral suppression) may also drive sample size, the cost benefits of longer MDRI HIAs may be limited. To test this, we calculated the impact of including sample size requirements for measuring viral suppression affected the survey sample size based on a longer MDRI HIA in a high incidence country and found that sample size decreases (approximately 80%) were retained. In addition, the expected cost savings over 2 to 3 years more than offset required investments to develop an improved HIA, making this an attractive public health investment. As these savings continue, more resources become available for investment in prevention activities likely to yield net savings of reduced downstream HIV/AIDS care and treatment costs. Additionally, an improved HIA would make surveillance using HIAs feasible in more countries and sub‐groups.

Sample sizes in surveys designed to estimate incidence can be reduced either by increasing the MDRI or by reducing the FRR, in an inter‐dependent manner. For example, keeping all the other parameters constant, the approximately 50% reduction in sample size achieved by an HIA with an MDRI of 240 days compared to 130 days if the FRR is held at 0.25% is approximately equivalent to that achieved if the FRR is reduced from 1% to 0% (45% reduction) if the MDRI is 130 days, or if the FRR is reduced from 2% to 0% (46% reduction) if the MDRI is 240 days. Our focus on longer MDRI is largely driven by the greater likelihood of a new HIA being able to reach this target compared to a FRR of 0%, which represents the absolute best performance achievable for this parameter, and by the fact that the MDRI is an inherent property of the HIA (albeit influenced by viral subtype) while FRR is highly context‐dependent (strongly affected by the proportion of people on ART with suppressed VL, or the number of elite controllers, for example). Controlling for context‐specific FRRs requires complex algorithms of multiple tests under conditions not intended by the assay manufacturers (e.g. alternative thresholds) 30. Nonetheless, it may be theoretically possible that a new HIA with low/zero FRR could also lead to cost savings due to reduced survey sample sizes, compared to HIAs with higher FRR.

Our market assessment has several limitations. First, our measures of 2014 and estimated 2015 to 2017 HIA use depend on reporting from many individuals and data sources. It is likely that we missed some HIA use, as we were only able to obtain sales figures from one of two primary manufacturers and unable to contact sources in several countries. Our projections for the various scenarios for 5 to 10‐year future use are based on the opinions of interview respondents and are necessarily subjective. We also assumed that some current usage, such as PHIAs and Demographic and Health Surveys generally conducted every 4 to 5 years per country, would continue into the future. Our projections for Scenarios 3a and 3b are based on the assumption that HIV diagnostic tests including a recency indicator can be sold at a price feasible for worldwide use. Furthermore, potential savings from an improved HIA will not accrue to the same institution that funds the development of a new assay, perhaps necessitating development costs to be borne by a government or foundation. Our cost estimates are based upon the two use cases where there are potential sample size reductions. We have not assessed how these potential savings could differ in a country with a concentrated versus generalized epidemic, as our focus was the global market. Lastly, although economic benefit is realized by reductions in survey sample size using HIAs with longer MDRI, another benefit would be to improve the precision of incidence estimates while maintaining current survey sample sizes. In this situation, the benefit of improved HIAs is the precision of the incidence estimates achievable rather than cost savings.

## 5 Conclusions

Thirty‐five years into the HIV epidemic, with approximately 2 million incident HIV infections annually, the need for a precise and time‐ and cost‐efficient method for measuring HIV incidence remains high. Substantial progress has been made as current RITAs, especially those using avidity tests and VL testing, meet many characteristics of target product profiles. Nevertheless, continued work is necessary to develop improved HIAs/RITAs that can provide more precise estimates with smaller sample sizes, including in populations with high antiretroviral use. Development and regulatory approval for HIAs/RITAs that can be used for individual patient management would vastly increase the market demand. Thus, ongoing efforts to improve HIA technology remain an important goal for the prevention of HIV infection globally.

## Competing interests

None of the authors have any conflicts of interest to declare.

## Authors’ contributions

CM, RH and MA designed the study. JO, PD, NP, SO and TM reviewed and provided substantive comments on the study design. CM, RH, NM, PS and JT collected and analyzed the data. CM, RH and NM wrote the initial draft of the manuscript, and all authors reviewed and provided substantive comments on the draft.

## Supporting information


**Table S1.** Eight main use cases for HIV incidence assays (HIAs).
**Table S2.** Organization types and numbers of interviewees.
**Table S3.** Estimated costs of assay development from transfer to delivery by development phase.
**Table S4.** Cost Breakdown for Surveillance Surveys – Fixed versus Variable Costs by Type of Resource.
**Table S5.** Detailed Breakdown of Population Based and Sentinel Surveillance surveys by Country and Region.
**Table S6.** Potential annual HIV infections averted if surveillance survey savings invested in cost‐effective HIV prevention interventions by use case and time frame (using base case savings from Table 4).
**Table S7.** Table of Data Elements used in Estimation of Costs and Cost Savings with Baseline Values (ranges if applicable) and Sources.
