# Supplementary material for: Assays for estimating HIV incidence: updated global market assessment and estimated economic value
Source: J Int AIDS Soc. 2017 Nov 22;20(3):e25018. doi: 10.1002/jia2.25018 (PMC5810336; doi:10.1002/jia2.25018)
Supplement: Supplementary file 1 — Table S1. Eight main use cases for HIV incidence assays (HIAs). Table S2. Organization types and numbers of interviewees. Table S3. Estimated costs of assay development from transfer to delivery by development phase. Table S4. Cost Breakdown for Surveillance Surveys – Fixed versus Variable Costs by Type of Resource. Table S5. Detailed Breakdown of Population Based and Sentinel Surveillance surveys by Country and Region. Table S6. Potential annual HIV infections averted if surveillance survey savings invested in cost‐effective HIV prevention interventions by use case and time frame (using base case savings from Table 4). Table S7. Table of Data Elements used in Estimation of Costs and Cost Savings with Baseline Values (ranges if applicable) and Sources. [file JIA2-20-e25018-s001.docx]

*Table S1. Eight main use cases for HIV incidence assays (HIAs)*

|  | Use | Description of Use |
| --- | --- | --- |
| **Uses related to estimating incidence** | National surveillance^a^ | To provide national estimate of incidence; may be part of a broader demographic study |
|  | Program, prevention, or trial planning | To provide incidence estimate for planning, prioritizing, or other instances when an estimate of incidence is required. Often may be for only a city or region (Example: prioritize programs or investments, or identify sites for intervention trials) |
|  | Key or sentinel populations | To provide incidence estimates in special sub-population using targeted sampling methods^b^ |
|  | Impact assessment^a^ | To assess the impact of a population-level intervention (e.g., community-level intervention) by comparing incidence before and after the intervention |
| **Uses NOT related to estimating incidence** | Case-based surveillance^b^ | To provide national or regional incidence estimates via case-based reporting of newly identified HIV+ individuals^c^ |
|  | Research purposes | Multiple potential applications (e.g., recruitment of recently infected individuals into longitudinal cohort studies) |
|  | Individual patient management | To guide clinical management and/or public health programs (e.g., selecting therapy, and/or prioritizing contact tracing) |
|  | Targeted prevention planning | To provide population-level data on recent infections to enable risk factors analysis or identify hot-spots to inform targeted prevention planning |

^a^ Probability sampling methods

^b^ Non-probability sampling methods

^c^ Testing alone is not used to obtain an incidence estimate, though recency test results incorporated into modeling has been used to extrapolate incidence estimates; methodologies vary greatly by country

*Table S2.* *Organization types and numbers of interviewees*

| Organization Types | N |
| --- | --- |
| Guidance body | 3 |
| Funder | 6 |
| Clinician | 2 |
| Developer | 2 |
| Manufacturer | 3 |
| User | 7 |
| Regulator | 1 |
| Surveillance | 3 |

*Table S3. Estimated costs of assay development from transfer to delivery by development phase*

| Phase | Duration  (months)^a^ | Developer Labor (full-time equivalents) | Cost Estimate  (USD)^b^ |
| --- | --- | --- | --- |
| Technical feasibility | 3 | 1.17 | 262,500 |
| Development | 21 | 6.98 | 2,021,250 |
| Product transfer | 16 | 3.28 | 736,875 |
| External evaluation | 5 | n/a | 200,000 |
| Total | 36 | 11.43 | 3,220,625 |

^a^ Number of months sum to greater than 36 because some phases will
take place concurrently

^b^ Cost estimates include labor valued at $225,000 per FTE plus supplies,
equipment, and fees for evaluation (see Table S7 for details)

*Table S4. Cost Breakdown for Surveillance Surveys - Fixed vs. Variable Costs by Type of Resource*

| Resource | Population-Based Survey | | Key Population Survey | |
| --- | --- | --- | --- | --- |
|  | Fixed (USD) | Variable (USD) | Fixed (USD) | Variable (USD) |
| Labor | 67,039 | 40,005 | 37,898 | 5,467 |
| Supplies | 8,264 | 208,045 | 1,752 | 68,655 |
| Transportation | 17,446 | 81,779 | 14,745 | 26,987 |
| Trainings | 53,646 | 0 | 17,703 | 0 |
| Other Direct Costs | 6,886 | 16,564 | 6,288 | 3,288 |
| Overheads | 55,319 | 53,442 | 30,244 | 19,980 |
| Total | 208,600 | 399,835 | 108,630 | 124,377 |
| per person | n/a | 182 | n/a | 71 |

*Table S5. Detailed Breakdown of Population Based and Sentinel Surveillance surveys by Country and Region*

| **Region** | **Country/region** | **Use Case** | **Year** | **Number of HIAs^a^** | **Prevalence** | **Sample Size** |
| --- | --- | --- | --- | --- | --- | --- |
| Africa | Malawi (PHIA)^b^ | population based survey | 2015-2017 avg | 2,500 | 10.7 | 23,364 |
| Africa | Zimbabwe (PHIA)^b^ | population based survey | 2015-2017 avg | 2,500 | 15.2 | 16,447 |
| Africa | Zambia (PHIA)^b^ | population based survey | 2015-2017 avg | 2,500 | 13.3 | 18,797 |
| Africa | Nigeria/Kaduna State | population based survey | 2015-2017 avg | 446 | 7.6 | 5907 |
| Africa | Nigeria/Akwa Ibom | population based survey | 2015-2017 avg | 690 | 7.6 | 9139 |
| Africa | Namibia (PHIA)^b^ | population based survey | 2015-2017 avg | 2,500 | 14 | 17,857 |
| Africa | Mozambique | population based survey | 2015-2017 avg | 1,200 | 11.1 | 10,811 |
| Africa | Cote d'Ivoire (PHIA)^b^ | population based survey | 2015-2017 avg | 2,500 | 3.7 | 40,000^c^ |
| Africa | Cameroon (PHIA)^b^ | population based survey | 2015-2017 avg | 2,500 | 4.3 | 40,000 ^c^ |
| Africa | Uganda (PHIA)^b^ | population based survey | 2015-2017 avg | 2,500 | 7.3 | 34,247 |
| Africa | Tanzania (PHIA)^b^ | population based survey | 2015-2017 avg | 2,500 | 5.1 | 40,000 ^c^ |
| Africa | Kenya (PHIA)^b^ | population based survey | 2015-2017 avg | 2,000 | 5.6 | 35,714 |
| Africa | Lesotho (PHIA)^b^ | population based survey | 2015-2017 avg | 2,500 | 23 | 10,870 |
| Africa | Swaziland (PHIA)^b^ | population based survey | 2015-2017 avg | 2,500 | 32.1 | 7788 |
| Africa | South Africa (HSRC)^d^ | population based survey | 2015-2017 avg | 3,250 | 12.6 | 25,794 |
| African | Benin | Sentinel Surveillance | 2013-2014 | 300 | 20 | 948 |
| African | Burkina Faso | Sentinel Surveillance | 2013-2014 | 100 | 3.6 | 669 |
| Pacific | China, Mainland | Sentinel Surveillance | 2013-2014 | 5,300 | 4.3 | 105,680 |
| Pacific | China, Mainland | Sentinel Surveillance | 2013-2014 | 500 | 7.6 | 5389 |
| Pacific | China, Mainland | Sentinel Surveillance | 2013-2014 | 100 | 2.1 | 94 |
| Pacific | China, Mainland | Sentinel Surveillance | 2013-2014 | 100 | 5.7 | 140 |
| Pacific | China, Mainland | Sentinel Surveillance | 2013-2014 | 300 | 3.2 | 7123 |
| Pacific | China, Mainland | Sentinel Surveillance | 2013-2014 | 100 | 2.1 | 94 |
| Pacific | China, Mainland | Sentinel Surveillance | 2013-2014 | 100 | 5.7 | 140 |
| Pacific | China, Mainland | Sentinel Surveillance | 2013-2014 | 100 | 3.9 | 1076 |
| Pacific | China, Mainland | Sentinel Surveillance | 2013-2014 | 100 | 5.3 | 571 |
| Pacific | China, Taiwan | Sentinel Surveillance | 2013-2014 | 100 | 4.5 | 1191 |
| Americas | Dominican Republic | Sentinel Surveillance | 2013-2014 | 100 | 4.8 | 104 |
| Southeast Asian | India | Sentinel Surveillance | 2013-2014 | 100 | 3 | 755 |
| Southeast Asian | India | Sentinel Surveillance | 2013-2014 | 1,400 | 9.5 | 12,022 |
| Southeast Asian | India | Sentinel Surveillance | 2013-2014 | 3,400 | 20.1 | 14,481 |
| Southeast Asian | India | Sentinel Surveillance | 2013-2014 | 300 | 2.5 | 6918 |
| Southeast Asian | India | Sentinel Surveillance | 2013-2014 | 100 | 11.8 | 458 |
| Southeast Asian | Indonesia | Sentinel Surveillance | 2013-2014 | 100 | 9.7 | 124 |
| Southeast Asian | Indonesia | Sentinel Surveillance | 2013-2014 | 100 | 2.3 | 128 |
| African | Malawi | Sentinel Surveillance | 2013-2014 | 100 | 7.1 | 99 |
| Mediterranean | Morocco | Sentinel Surveillance | 2013-2014 | 100 | 0.9 | 927 |
| Mediterranean | Morocco | Sentinel Surveillance | 2013-2014 | 100 | 1.5 | 324 |
| Mediterranean | Morocco | Sentinel Surveillance | 2013-2014 | 100 | 1.7 | 359 |
| Mediterranean | Morocco | Sentinel Surveillance | 2013-2014 | 100 | 5.1 | 372 |
| African | South Africa | Sentinel Surveillance | 2013-2014 | 800 | 6.8 | 9998 |
| African | South Africa | Sentinel Surveillance | 2013-2014 | 1,500 | 4.6 | 27,937 |
| African | Togo | Sentinel Surveillance | 2013-2014 | 100 | 10 | 330 |
| African | Togo | Sentinel Surveillance | 2013-2014 | 100 | 0.6 | 329 |
| African | Uganda | Sentinel Surveillance | 2013-2014 | 100 | 11.5 | 625 |
| African | Uganda | Sentinel Surveillance | 2013-2014 | 100 | 12.1 | 512 |
| African | Uganda | Sentinel Surveillance | 2013-2014 | 500 | 4.4 | 8527 |
| Pacific | Vietnam | Sentinel Surveillance | 2013-2014 | 100 | 5.1 | 256 |

^a^Number of samples tested.

^b^Population-based HIV impact assessments

^c^Sample size for population-based surveys is capped at 40,000 based on feasible sample size for national HIV surveillance surveys

^d^Human Sciences Research Council

*Table S6. Potential annual HIV infections averted if surveillance survey savings invested in cost-effective HIV prevention interventions by use case and time frame (using base case savings from Table 4)*

| Use Case Description | Next 2-3 years | | | Next 5-10 years (Scenario 2) | | |
| --- | --- | --- | --- | --- | --- | --- |
|  | **MDRI: 154 days** | **MDRI: 240 days** | **MDRI: 280 days** | **MDRI: 154 days** | **MDRI: 240 days** | **MDRI: 280 days** |
| Population-Based Surveys | 75,187 | 208,945 | 239,684 | 101,502 | 282,075 | 323,573 |
| Key Population Surveillance Surveys | 16,724 | 47,853 | 55,107 | 22,577 | 64,602 | 74,394 |
| Total | 91,911 | 256,798 | 294,791 | 124,079 | 346,677 | 397,967 |

*Table S7: Table of Data Elements used in Estimation of Costs and Cost Savings with Baseline Values (ranges if applicable) and Sources*

| Category | Data Element | Baseline Value (USD) | low | high | Source |
| --- | --- | --- | --- | --- | --- |
| *Assay Development Costs* | | | | | |
|  | Annual Labor Cost per FTE (fully loaded) | 225,000 |  |  | Author’s estimate verified by industry experts |
|  | CEPHIA Development & Qualification Panels during assay development | 150,000 |  |  | Author’s estimate verified by industry experts |
|  | CEPHIA Evaluation Panel (specimens & kits) | 200,000 |  |  | Author’s estimate verified by industry experts |
|  | Additional Capital Equipment required during assay development | 300,000 | 100,000 | 300,000 | Author’s estimate verified by industry experts |
| *Surveillance Survey Costs* | | | | | |
| Population-based surveys | |  |  |  |  |
|  | Fixed Costs (per survey) | 208,600 |  |  | Author's calculation based upon IBBS surveys conducted in Botswana & Ghana |
|  | Variable costs (per sample) | 182 |  |  | Author's calculation based upon IBBS surveys conducted in Botswana & Ghana |
| Key Population surveys | |  |  |  |  |
|  | Fixed Costs (per survey) | 108,630 |  |  | Author's calculation based upon IBBS surveys conducted in Botswana & Ghana |
|  | Variable costs (per sample) | 71 |  |  | Author's calculation based upon IBBS surveys conducted in Botswana & Ghana |
| *Sample Size Reductions possible from lengthened MDRI* | | | | | |
| Population-based surveys | |  |  |  |  |
|  | Lengthen MDRI from 130 - 154 days | 14.5% | 9.1% | 18.1% | Author’s calculation based upon [[11](#_ENREF_11)] |
|  | Lengthen MDRI from 130 - 240 days | 40.2% | 25.2% | 50.3% | Author’s calculation based upon [[11](#_ENREF_11)] |
|  | Lengthen MDRI from 130 - 280 days | 46.2% | 28.9% | 57.7% | Author’s calculation based upon [[11](#_ENREF_11)] |
| Key Population surveys | |  |  |  |  |
|  | Lengthen MDRI from 130 - 154 days | 13.3% | 8.3% | 16.6% | Author’s calculation based upon [[11](#_ENREF_11)] |
|  | Lengthen MDRI from 130 - 240 days | 38.0% | 23.8% | 47.5% | Author’s calculation based upon [[11](#_ENREF_11)] |
|  | Lengthen MDRI from 130 - 280 days | 43.8% | 27.4% | 54.7% | Author’s calculation based upon [[11](#_ENREF_11)] |
| *Annual cost to avert one HIV infection through cost-effective HIV prevention interventions* | | | | | |
|  | Annual cost to avert one infection assuming most cost-effective interventions in Africa region (mass media & peer education with STI treatment of sex workers, condom promotion) | $58.00 | $57.80 | $300 | Author's calculation based upon [[12](#_ENREF_12), [13](#_ENREF_13)] |
| *Annual Public Health Value of an averted HIV infection* | | | | | |
|  | Annual value of lifetime cost of HIV treatment discounted at 3% over 10 years | $612.00 |  |  | Author's calculation based upon [[12](#_ENREF_12)] |
